# Characterization and Correction of Geometric Distortions in 814 Diffusion Weighted Images

**DOI:** 10.1371/journal.pone.0152472

**Published:** 2016-03-30

**Authors:** Jeffrey Mark Treiber, Nathan S. White, Tyler Christian Steed, Hauke Bartsch, Dominic Holland, Nikdokht Farid, Carrie R. McDonald, Bob S. Carter, Anders Martin Dale, Clark C. Chen

**Affiliations:** 1 School of Medicine, University of California San Diego, San Diego, California, United States of America; 2 Center for Theoretical and Applied Neuro-Oncology, University of California San Diego Moores Cancer Center, San Diego, California, United States of America; 3 Multimodal Imaging Laboratory, University of California San Diego, San Diego, California, United States of America; 4 Neurosciences Graduate Program, University of California San Diego, San Diego, California, United States of America; 5 Department of Radiology, University of California San Diego, San Diego, California, United States of America; 6 Department of Psychiatry, University of California San Diego, San Diego, California, United States of America; 7 Department of Neurosurgery, University of California San Diego, San Diego, California, United States of America; University of Pécs Medical School, HUNGARY

## Abstract

**Introduction:**

Diffusion Weighted Imaging (DWI), which is based on Echo Planar Imaging (EPI) protocols, is becoming increasingly important for neurosurgical applications. However, its use in this context is limited in part by significant spatial distortion inherent to EPI.

**Method:**

We evaluated an efficient algorithm for EPI distortion correction (EPIC) across 814 DWI scans from 250 brain tumor patients and quantified the magnitude of geometric distortion for whole brain and multiple brain regions.

**Results:**

Evaluation of the algorithm’s performance revealed significantly higher mutual information between T1-weighted pre-contrast images and corrected b = 0 images than the uncorrected b = 0 images (*p* < 0.001). The distortion magnitude across all voxels revealed a median EPI distortion effect of 2.1 mm, ranging from 1.2 mm to 5.9 mm, the 5^th^ and 95^th^ percentile, respectively. Regions adjacent to bone-air interfaces, such as the orbitofrontal cortex, temporal poles, and brain stem, were the regions most severely affected by DWI distortion.

**Conclusion:**

Using EPIC to estimate the degree of distortion in 814 DWI brain tumor images enabled the creation of a topographic atlas of DWI distortion across the brain. The degree of displacement of tumors boundaries in uncorrected images is severe but can be corrected for using EPIC. Our results support the use of distortion correction to ensure accurate and careful application of DWI to neurosurgical practice.

## Introduction

Diffusion Weighted Imaging (DWI) is increasingly used to guide the clinical management of neurosurgical brain tumor patients due to the unique ability of this technique to identify regions of high cellularity within tumor and to estimate the integrity of adjacent white matter [1–5]. As a result, three powerful applications of DWI in neurosurgery are target selection for stereotactic biopsy [4], estimation of tumor pathology [6–9], and visualization of fiber tracts during surgical planning [2,3,5]. However, accurate neurosurgical application of DWI is limited by geometric distortion inherent to the imaging modality [10]. Methods for accurate and efficient distortion correction may afford opportunities to maximize the potential of DWI-assisted neurosurgical practice.

The fundamental principle that allows for DWI is the quantification of water diffusion using rapid MR image acquisition [11]. In most clinical applications, this acquisition is achieved through Echo Planar Imaging (EPI), where entire images are acquired in single shots by rapidly reversing the frequency-encoding gradient [12]. While the technique affords high temporal resolution, it has the undesirable effect of introducing significant geometric distortion and intensity variation that result from the interaction between the main magnetic field of the scanner and the patient’s anatomy [10,13]. While previous methods have been reported to correct these effects, many are computationally intensive, requiring more time and additional expertise [10,13–21].

We previously reported a method for correcting for such distortions by utilizing an additional non-diffusion weighted (b = 0) DWI volume with reversed phase encoding polarity, requiring only seconds of additional scan time [19]. This additional image possesses equal and opposite distortion magnitude in the plane of acquisition, which can be used to determine regional spatial distortion as the basis for distortion correction. Since its publication, this method has been employed routinely in our clinical and research protocols and thus far has had its clinical utility demonstrated in prostate [22] and breast cancer imaging [23]. Here, we report the application of this method to images from a large cohort of neurosurgical patients with various brain tumors. By mapping the distribution of geometric distortion to a standard brain, our study demonstrates the importance of utilizing distortion correction in neurosurgical applications of DWI.

## Methods

### Patients

A total of 250 patients with intra-cranial neoplasms at the University of California, San Diego (UCSD) Health Care System were identified retrospectively. The study was approved by the UCSD Institutional Review Board and all participating patients signed informed consent. Imaging was collected beginning in 2010 and at the time of analysis, 822 imaging series were available. Inclusion in the study required a DWI in addition to artifact free T1-weighted structural images with and without contrast obtained during the imaging session. Eight imaging series were excluded from this study due to excessive imaging artifacts attributable to motion.

### Data acquisition

All MR imaging was performed on a 3T Signa Excite HDxt scanner (GE Healthcare, Milwaukee, Wisconsin). As part of the standard protocol we included a 3D volumetric T1-weighted inversion recovery spoiled gradient-echo sequence pre- and post-gadolinium (TE = 2.8 ms, TR = 6.5 ms, TI = 450 ms, flip angle = 8°, FOV = 24 cm; 0.93 x 0.93 x 1.2 mm). Our diffusion protocol consisted of a single-shot pulsed-field gradient Stejskal-Tanner EPI sequence with 4 b-values (b = 0, 500, 1500, and 4000 s/mm^2^) and 1, 6, 6, and 15 unique diffusion directions, respectively (TE = 96 ms, TR = 17 s, flip angle = 8°, FOV = 24 cm; 1.875 x 1.875 x 2.5 mm).

### Preprocessing

DWI and T1-weighted images were corrected for gradient magnetic field nonlinearity using previously described methods [24]. Diffusion images were additionally corrected for motion and eddy currents [25], and registered to the pre-gadolinium T1-weighted image (T1W) using a rigid-body transform. An inverse transform was applied to resample the T1W to diffusion native space as a basis for evaluation of our algorithm. Additionally, each T1W was registered to a template normal T1W (S1 File) using a rigid-body transform in order to create transforms necessary to place the diffusion data and displacement maps in a common template space. Each registration was manually reviewed to assess accurate alignment.

### Distortion correction and quantification

**Fig 1:**
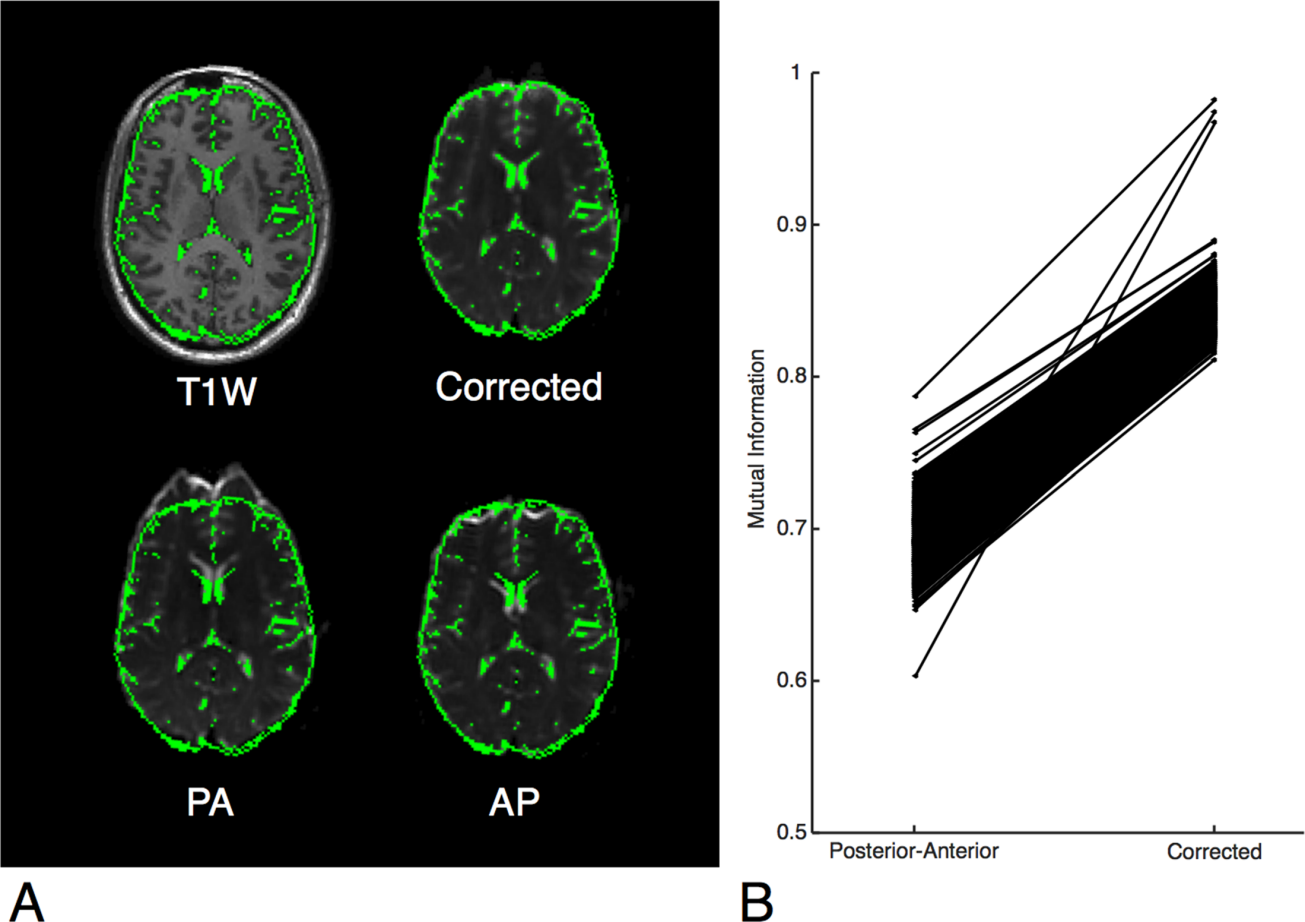
**A:** Demonstration of the algorithm on a single subject. A mask of the CSF created from the T1W is shown overlaid in green. Top left, subject’s T1-weighted MRI image. Top right, subject’s corrected b = 0 image. Bottom, subject’s uncorrected b = 0 image in both encoding directions is shown, posterior-anterior (PA) and anterior-posterior (AP). The CSF is shown to fit the boundaries of the corrected b = 0 image but not the uncorrected images. This effect is most pronounced in the frontal lobe and anterior aspect of the lateral ventricles. **B:** Mattes mutual information calculated between the T1W and b = 0 images. In all 814 imaging series, the corrected b = 0 image was more similar to the T1W image than the uncorrected b = 0 image.

Correction of the patient-specific distortions particular to EPI was performed using the EPI correction algorithm (EPIC) previously described by Holland et al (2010). Briefly, this method utilizes the symmetry of the distortions arising from opposite phase encoding polarities to determine the distortion field that maps voxels back to their true locations (Fig 1A). During acquisition of DWI scans, two non-diffusion-weighted (b = 0) volumes with opposite phase encoding polarities were acquired at the beginning of the scan and used to estimate the voxel-wise distortion field in the anterior-posterior direction (axial plane). The resulting distortion maps were then applied to the remainder of each subjects’ diffusion data and transformed to template space.

In order to assess the effectiveness of the algorithm, the Mattes mutual information (MI) similarity metric [26] was used. This histogram-based measure of similarity has been used commonly in neuroimaging to compare images of different modalities [27–29]. This measure was calculated between the T1W in diffusion space and corrected b = 0 and compared to that calculated from the uncorrected posterior-anterior encoded b = 0 scan using a Wilcoxon signed rank test.

To quantify the magnitude of geometric distortion as a function of anatomic location, a cohort-wide displacement atlas was generated by averaging the displacement at each voxel across all registered displacement maps (S2 File).

## Results

### Algorithm evaluation

**Fig 2:**
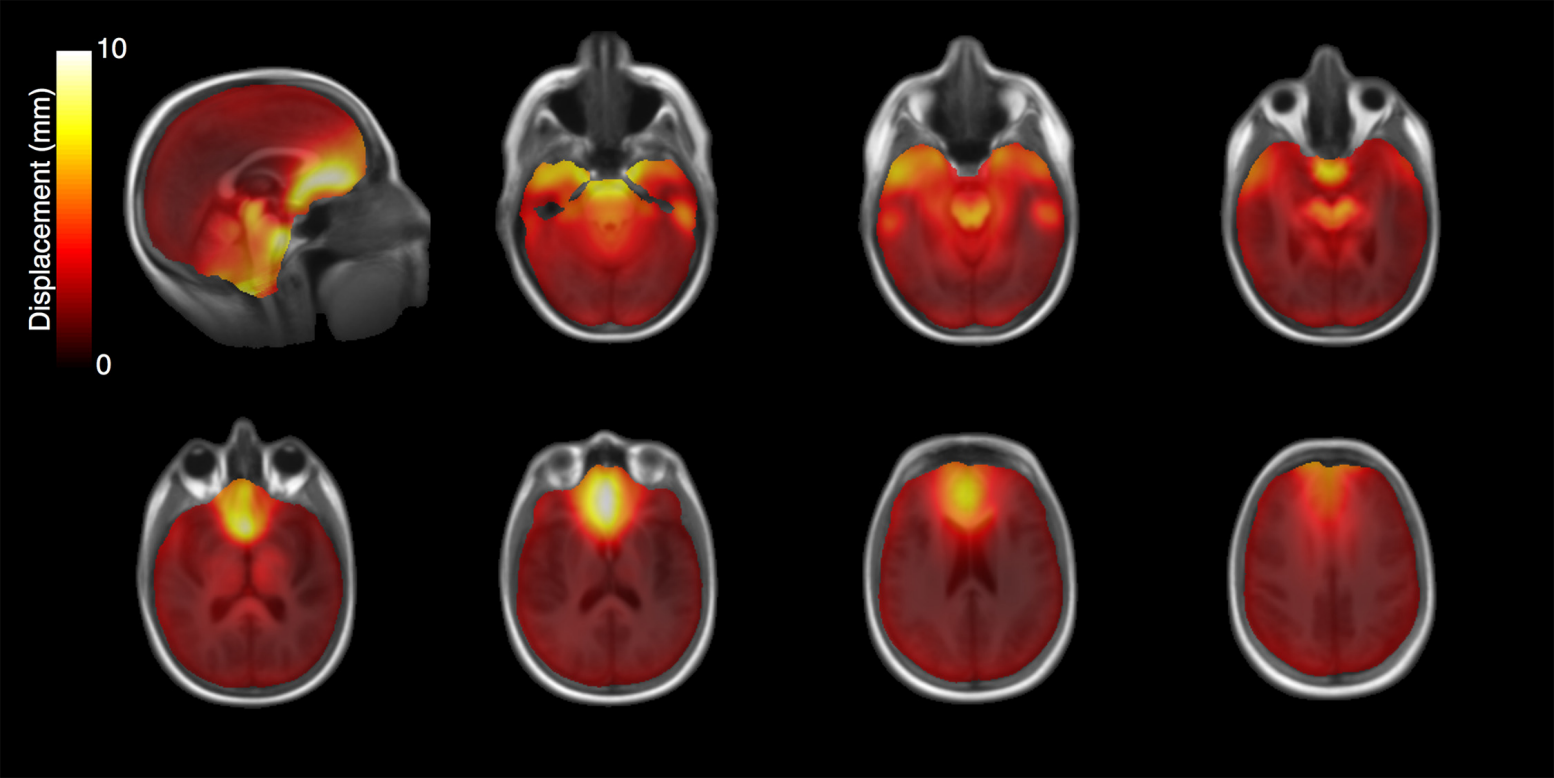
EPI distortion atlas. Average anterior-posterior displacement calculated per voxel over all 814 imaging series registered to a normal brain template.

**Fig 3:**
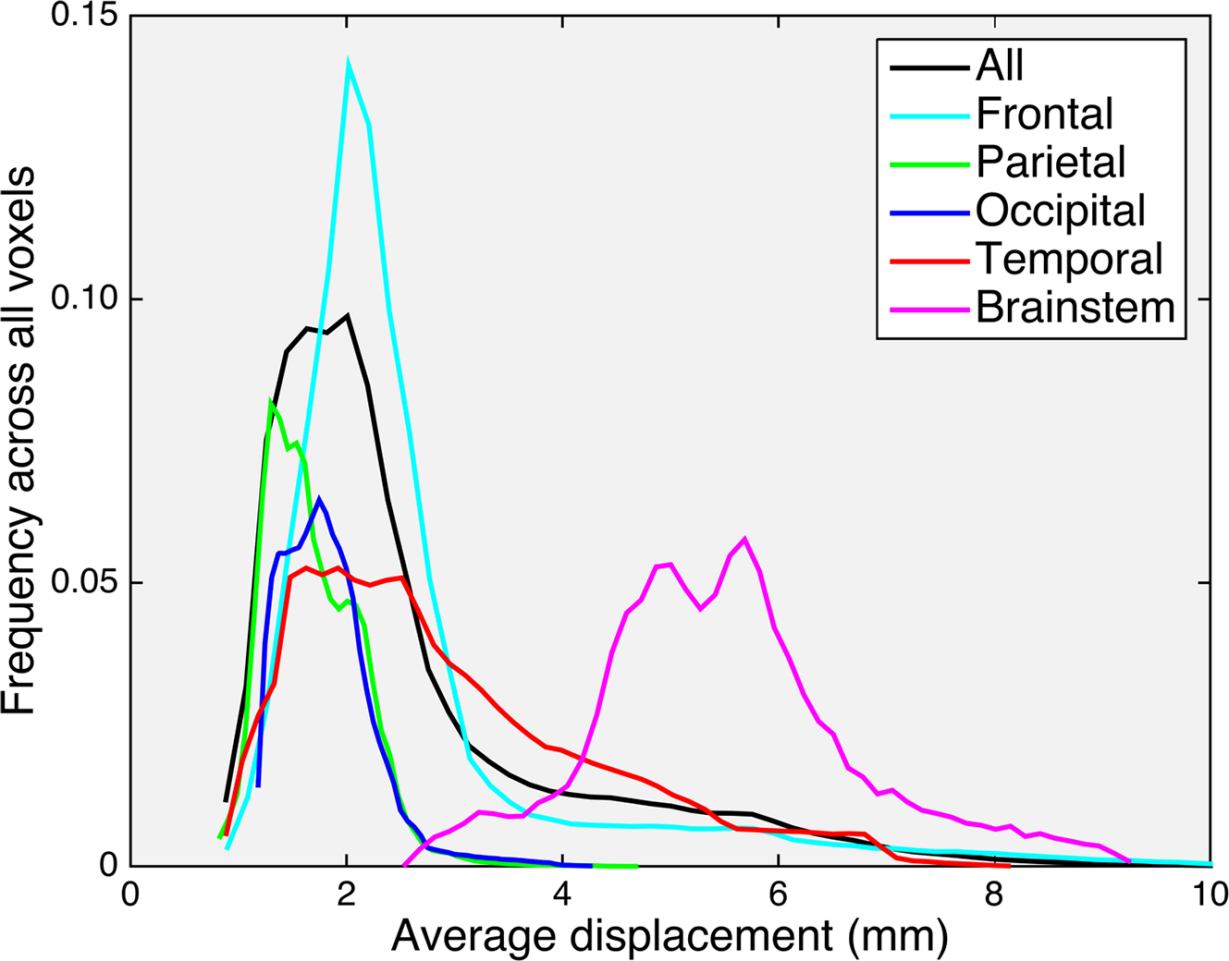
EPI distortion histograms. Anterior-posterior displacement across all voxels within the template brain (black), frontal lobe (cyan), parietal lobe (green), occipital lobe (blue), temporal lobe (red), and brainstem (magenta). Frequency is reported as the fraction of voxels in the region of interest undergoing a given displacement.

**Fig 4:**
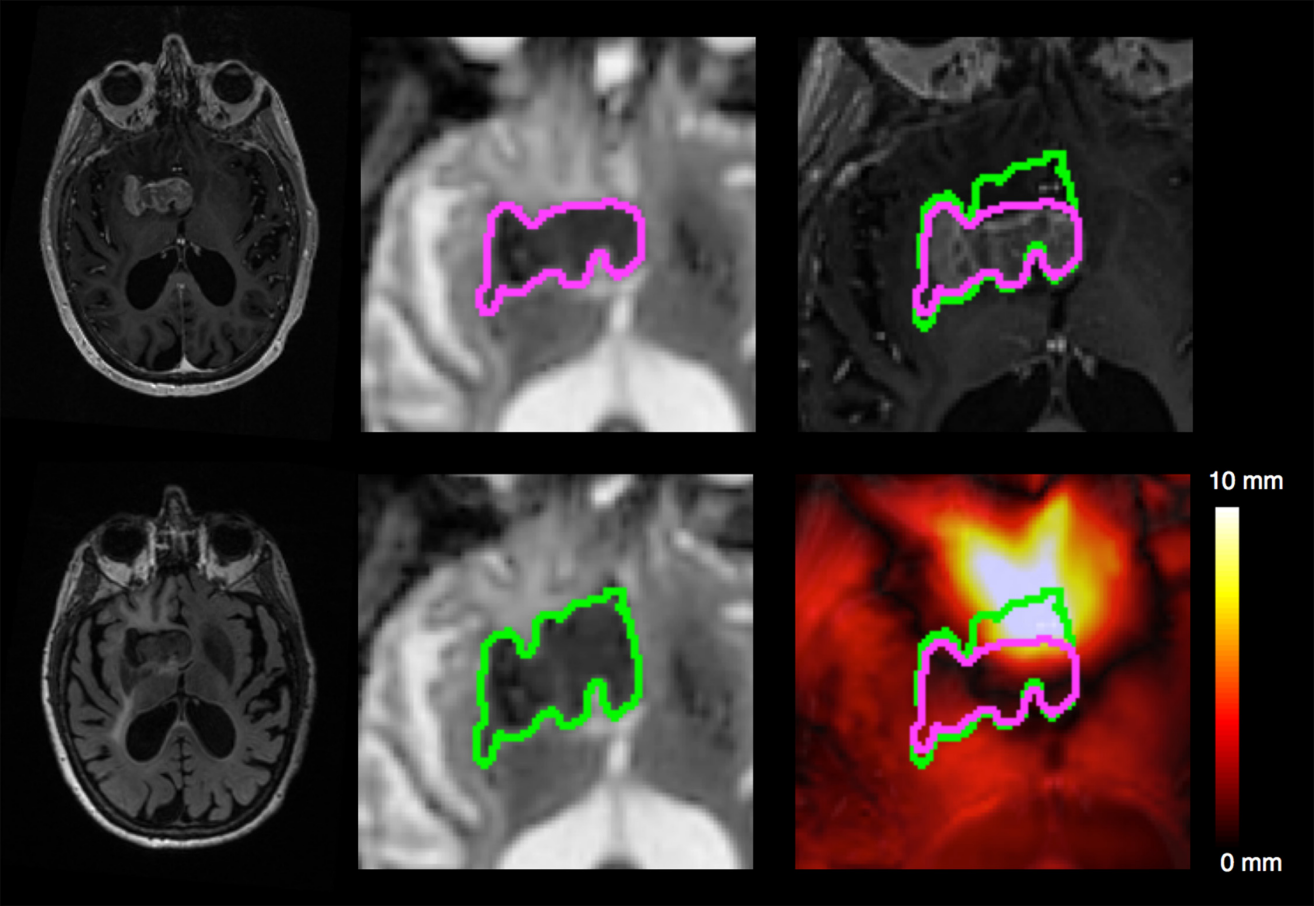
Illustrative example of a tumor’s displacement with and without correcting for EPI distortions. Top left, T1-weighted image with contrast. Bottom left, Fluid Attenuated Inversion Recovery image. Middle: ADC calculated from corrected diffusion image (top) and uncorrected diffusion image (bottom) with the regions of diffusion restriction outlined (green/purple). Top right, corrected (purple) and uncorrected diffusion restriction outline (green) overlaid on T1-weighted image with contrast. Bottom right, corrected and uncorrected diffusion restriction outline overlaid on the displacement map.

Comparisons of the MI similarity metric revealed a higher similarity between T1W and the corrected b = 0 image than the uncorrected b = 0 image in all 814 imaging series (p < 0.001, Fig 1B), indicating that every diffusion image in our cohort was more similar to the T1W after correcting for EPI distortions using EPIC. The displacement atlas displays the average spatial distortion at every voxel (Fig 2). This atlas revealed that the parenchyma adjacent to bone-air interfaces, including the orbitofrontal cortex and temporal pole, had distortions as severe as the brainstem. Quantitative analysis of the displacement atlas showed a median displacement of 2.11 mm, that ranged from 1.2 mm to 5.9 mm, the 5th and 95th percentile, respectively (Fig 3). The regions with the most severe distortions were the brainstem (median distortion d = 5.43 mm), temporal lobe (d = 2.61 mm), and frontal lobe (d *=* 2.21 mm), while the parietal (d = 1.61 mm) and occipital (d = 1.77 mm) lobes had the least amount of distortion (Fig 3). Within the frontal and temporal lobes, the parenchyma adjacent to the bone-air interfaces, including the orbitofrontal cortex and temporal pole, had distortions as severe as the brainstem (Fig 2). We sought to visualize the impact of DWI related spatial distortion in neurosurgical navigation. To this end, we selected a patient with a tumor with clearly visible boundaries on DWI that extends to tissue adjacent to one of the major bone-air interfaces. To delineate the tumor boundary on diffusion images and assess the effect of image distortion, apparent diffusion coefficient (ADC) maps were generated from the uncorrected and corrected diffusion images using the Stejskal-tanner equation with the diffusion images of the longest diffusion time (b = 4000). As shown in Fig 4, the size of the lesion was artificially increased in the uncorrected ADC image when compared to T1W and Fluid Attenuated Inversion Recovery images while the corrected ADC image more accurately estimated the boundaries of the tumor.

## Discussion

Geometric distortion associated with standard DWI sequences has been characterized previously, and poses a significant challenge to achieving high accuracy in DWI-based neurosurgical applications [10,13–19,30]. Here, we assess the significance, applicability, and performance of DWI spatial distortion correction in the context of neurosurgical planning, employing EPIC, a method previously published by our laboratory [19]. We demonstrate that the application of EPIC results in diffusion images considerably closer to the patient’s true anatomy as indicated by T1W. Importantly, the method was integrated into our standard MR clinical protocols, requiring only a single extra image sequence lasting fewer than 5 seconds. The additional short scan time required for EPIC did not adversely impact clinical work-flow and was simple to implement.

Analysis of this large neurosurgical data set of 814 DWIs afforded an opportunity to generate an anatomic atlas of DWI related distortion effects. While DWI distortion affects the entire brain, it is well known [10] (and can be seen from our analysis) that the distortions are most severe in the regions adjacent to bone-air boundaries such as the orbitofrontal cortex, temporal pole, and brain stem. These regions have particular significance for neurosurgeons, as they are common sites for primary brain tumors [31]. We demonstrate that the magnitude of DWI distortion could significantly impact surgical planning and guided stereotactic biopsy by grossly distorting tumor borders as seen in an example subject (Fig 4). These effects may be even more pronounced in other applications of diffusion imaging to neurosurgery such as tractography [32]. The implications of these results together indicate that use of DWI in neurosurgical applications without accurate distortion correction methods like EPIC, may contribute to suboptimal surgical outcomes and ultimately impact the quality of patient care.

There are two potential limitations to our study. First, while 814 DWI images were employed to assess the applicability of distortion correction using EPIC in neurosurgical planning, it is important to note that this study represents a single institutional experience from one scanner. Differences in scanners including hardware, field strength, software version, and shimming can produce significant variation in EPI distortions between institutions. As such, validation of our results by independent institutions is warranted. Second, the integrated sequence used for EPIC is a non-standard prototype used for research that is currently not available commercially. Nevertheless, the sequence requires only an additional scan time of <5 seconds and can be easily integrated into a clinical work flow. While the results reported here are pertinent to DWI sequences, our method can be applied to other EPI based imaging, including perfusion imaging and functional MRI.

## Conclusions

DWI is a powerful modality that adds information beyond that afforded by conventional MR imaging. While DWI holds promise to offer benefits to neurosurgical practice, it is currently limited by imaging distortions that result from physical constraints of the image acquisition. Implementation of DWI distortion correction methods like EPIC may help facilitate the development of DWI assisted neurosurgical navigation, and may ultimately improve patient outcomes.

## Supporting Information

S1 File Normal T1-weighted template. (GZ)

S2 File EPI distortion atlas. Average anterior-posterior displacement (mm) calculated per voxel over all 814 imaging series registered to a normal brain template (S1 File).
(GZ) Click here for additional data file.
